# The history of smoking cessation support in Hungary: A narrative review

**DOI:** 10.18332/tpc/191141

**Published:** 2024-08-12

**Authors:** Zsuzsa Cselkó, Judit Tisza, Márta Fényes

**Affiliations:** 1 Department of Methodology, National Korányi Institute of Pulmonology, Budapest, Hungary

**Keywords:** smoking, cessation support, Hungary

## Abstract

Tobacco use remains the largest preventable cause of death worldwide, including Hungary with a smoking-related death rate in 2019 of 360/100000 (age-standardized death rate), among the highest in the World Health Organization (WHO) European Region. Despite the well-formulated tobacco control interventions defined by the WHO Framework Convention on Tobacco Control (WHO FCTC) in place, smoking prevalence in 2019 was high (27%) and has not decreased since 2014. Therefore, greater emphasis should be placed on addressing and treating smokers. This narrative review summarizes the progress in smoking cessation support in Hungary to identify strengths and areas for improvement. A literature search was conducted in the Hungarian Arcanum Digital Science Library. After 2012, data were derived from the National Methodology Center for Cessation Support reports. The National Korányi Institute for Pulmonology established the first organized network of cessation counselling services in 1987 in outpatient pulmonary clinics (OPCs) sponsored by a State Insurance tender. By 1999, individual behavioral counselling with medication was accessible to 130 healthcare providers, due to the support of pharmaceutical companies. Since 2005, the National Health Insurance Fund has financed smoking cessation support in OPCs, albeit at a low value. Having recognized that OPCs are overburdened by the organizational tasks of cessation support and that funding is intermittent, from 2020, the counselling service has transferred to the existing network of health promotion offices, although without specific funding for cessation programs and communication. Adequate and regular funding for established counselling services and nicotine withdrawal treatment is essential to achieve progress in tobacco control. The role of healthcare professionals is outstanding; therefore, individual responsibilities should be recognized.

## INTRODUCTION

Tobacco use remains the largest preventable cause of death worldwide, including Hungary with a smoking-related death rate in 2019 of 360/100000 (age-standardized death rate), among the highest in the World Health Organization (WHO) European Region^1^. Well-formulated tobacco control interventions defined by the WHO Framework Convention on Tobacco Control (WHO FCTC) are in place^2^. However, the prevalence of smoking in 2019 was high (27%) and did not decreased from 2014–2019^3^.

Owing to regulations aimed at curbing smoking and the population’s more health-conscious behavior, developed countries have significantly reduced the proportion of smokers^2^. Some argue that those unable to quit on their own in these societies are more likely to be highly addicted to nicotine or belong to a risk group where comorbidities or other factors such as lower socioeconomic status, impede successful quitting^4^. This is unlikely to be the case in Hungary for the following reasons. Smoking intensity has decreased over the past twenty years^5-8^. The association between education and smoking only strengthened in the case of those with primary school education, while the other categories remained almost unchanged over the past two decades. Similarly, the association between economic status and smoking, except for pensioners, weakened in all groups. More than half (54%) of smokers aged >18 years had already attempted to quit in 2019^5^, compared to 46% in 2000^6^. These indicate that the decline in the proportion of smokers in Hungary has not ceased because only a well-defined but more difficult-to-motivate risk group of people remain smokers. It should be noted that only 2% of smokers in Hungary sought professional help to quit smoking, and 14% said they would do so in the future^5^. Thus, among other tobacco control interventions, greater emphasis should be placed on addressing and treating smokers based on their age and health status to reduce the burden caused by smoking.

This review aims to summarize the evolution of smoking cessation support in Hungary, focusing on behavioral counselling as an evidence-based and effective treatment method to identify strengths and determine areas for improving cessation support services.

A literature search was conducted in May 2023 in the Hungarian Arcanum Digital Science Library using the term ‘smoking cessation’^9^. The Arcanum Digital Science Library is a database of digitalized periodicals initially published on paper in Hungary. It includes hundreds of national scientific and specialist periodicals, and daily and weekly newspapers. The search yielded 6694 results from 1868 to 2022. A total of 5323 results until December 2012 were considered for the analysis. After reviewing the content and deleting duplicates, 96 publications from the abovementioned categories containing specific information on smoking cessation support or behavioral counselling in Hungary were selected for analysis. Starting from 2013 or After 2012, data on smoking cessation were obtained from reports of the National Methodology Center for Cessation Support established in November 2012 and tasked with overseeing and coordinating smoking cessation activity in Hungary.

## SMOKING CESSATION SUPPORT IN HUNGARY

### The beginnings

**Figure 1:**
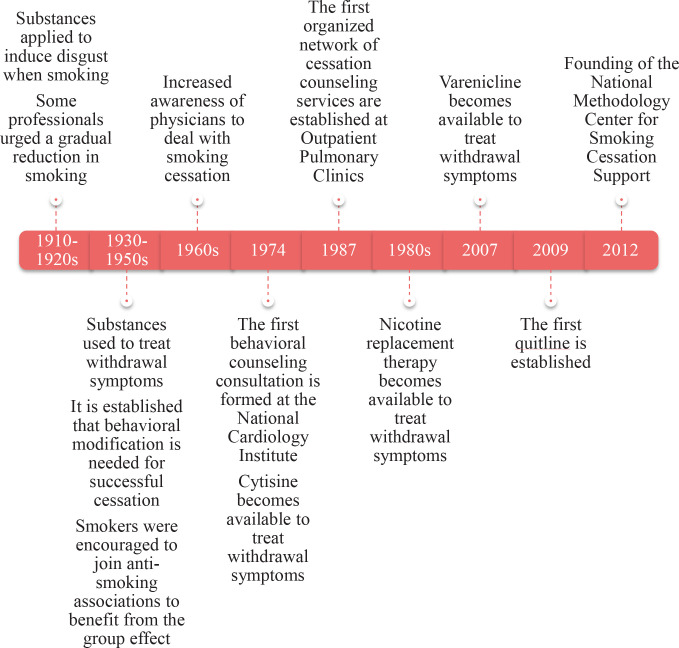
Milestones in the evolution of smoking cessation support in Hungary

The first community objections to smoking did not arise from health considerations but from social co-existence^10^. Although the earliest clues to the physiological underpinnings of tobacco use first came to light in the 1920s and the first major and almost conclusive evidence of the effects of smoking on health was published in 1950, there is indication that the harmful effects of smoking, especially the effects of nicotine, and cessation have preoccupied society and healthcare professionals since the early twentieth century in Hungary (Figure 1)^10-12^. During the 1910s–1920s, practices revolved around the application of substances (e.g. silver nitrate, tannic acid) in the mouth, which induced disgust when smoking^11,12^. However, the efficacy and ethicality of the process were questioned. Some professionals urged a gradual reduction in smoking^12^.

In the 1930s–1940s, it was acknowledged that to help smokers quit smoking, physicians needed much time, insight, certain psychoanalytical knowledge, and a supportive environment^12^. Withdrawal symptoms were first treated with stimulants (caffeine, theobromine) or sedatives (valium, sodium bromide), later with the injection of lobeline (partial nicotine agonist)^12,13^. The latter did not have any established benefits in cessation^14^. Smokers were also encouraged to join anti-smoking associations to benefit from the group effect^12^. The first known Hungarian written materials on smoking and quitting methods were published in 1938, and by 1944, it was established that behavioral modification was needed for successful cessation^15,16^.

Lobeline was still used during the 1960s, reportedly in smoking patients treated at Kékestet**ő** State Hospital, where patients with respiratory diseases, especially tuberculosis, were cared for^13^. Piperidinomethyl-cyclohexanon-2-chlorhydrate, under the name spiractin, was developed and produced by the Gedeon Richter Chemical Factory between 1958–1992 (known as K**ő**bánya Pharmaceutical Factory between 1948–1990). It was increasingly used instead of lobeline for respiratory stimulation in ambulances and to ease cessation^17,18^. Intramuscular injection of spiractin produced a serotonin-like effect^19^. However, apart from a Hungarian report, there is no record of its proven efficacy as a cessation aid.

### Increased awareness of physicians

Since the 1960s, physicians called on the healthcare workers’ union to organize campaigns for healthcare workers to quit smoking and to have medical schools include the health effects of smoking and cessation support in their curricula^20^. It became widely known and recognized that the first hospital for cessation support was successfully established in Stockholm, Sweden, in 1965^21^. Hungarian journals reported that Swedish experts developed the method for smoking cessation: 1) education about the harmful effects of smoking, 2) treatment of withdrawal symptoms (still with lobeline), 3) change in behavior patterns (i.e. finding new hobbies), and 4) prevention of relapse: daily consultations with the physician help find solutions to problems that arise with quitting^22^. At the same time, the tobacco industry’s harm reduction approach was already advertised as a solution to the smoking epidemic. A Hungarian-designed smoke filter cigarette holder called the ‘health protector holder’ was said to work based on aerodynamic principles by repeatedly accelerating, impacting, and cooling tobacco smoke particles; thus, it precipitates tar and nicotine, which are collected on the metal insert of the holder^23^. The holder was advertised using the exact wording known from the tobacco industry marketing strategy today, seen in ‘corporate social responsibility’ advertisements, claiming to reduce harm but aiming to promote tobacco consumption: ‘Those who have not started smoking should not start. Those who have already started should try to quit or decrease consumption. If someone cannot quit smoking, the Superfilt holder is a great way to reduce harm’^2,23,24^.

### The first behavioral counselling services formed

At the beginning of the 1970s, information on organized smoking cessation support activities (mostly group counselling) increased worldwide such as in Czechoslovakia, Germany, England, and the United States, urging healthcare professionals to emphasize the role of physicians in health promotion^25^. The Weekly Medical Journal reported that local initiatives to establish behavioral counselling services have been forming in Czechoslovakia for over a decade. It is said that such institutes should be established in every district and run by the head of the health education service. Physicians should practice brief intervention and refer patients to counselling services. However, self-volunteers would also be accepted. In addition to physicians, nurses with secondary education are also needed in such counselling centers. The requirements for the room and equipment are listed, and a detailed work plan is provided. At first, the patient is called for weekly counselling, then less frequently, and eventually followed for at least a year and a half ^26^.

In Hungary, a limited number of behavioral counselling consultations led by a physician or psychologist existed at the time. One such initiative started operation in 1974 at the National Cardiology Institute. Patients who suffered a heart attack were followed up three months after leaving the hospital, and if they were still smoking, they were offered group counselling^27^. Cytisine has been available since 1974 (initially without, later with reimbursement), and nicotine replacement therapy (NRT) from the beginning of the 1980s^28,29^. The Society Against Smoking was founded in 1975, emphasizing the role of teachers and healthcare professionals in preventing the further spread of smoking and serving as role models^29,30^. There were also examples of large companies offering help to employees to quit smoking through occupational medicine services, and workers’ unions were formed to combat smoking in the workplace^28^.

The first organized network of behavioral counselling services was established in 1987 in outpatient pulmonary clinics (OPCs) by successfully applying for a state insurance tender. The program was led by the Methodology Department of the National Korányi Institute for TB and Pulmonology (NKIP)^31^. In 1993, the program was revived by the National Health Protection Institute with the support of pharmaceutical companies. By 1999, the three-month individual behavioral counselling program (with a nine-month follow-up), including pharmacotherapy, was available in 130 OPCs, pulmonary and cardiology departments, and other outpatient clinics, where physicians and nurses offered counselling^32^. Healthcare workers were trained according to European guidelines under the leadership of the Hungarian Pulmonary Society^33^.

At the beginning of the 2000s, the legislator intended to strengthen the cessation counselling service based on the following pillars, but still without dedicated, predictable, and long-term funding^34^:

- Continuous public communication with media activities can be effective through brochures, websites, and toll-free numbers to provide advice on cessation possibilities.
- Testing and implementing comprehensive hospital anti-smoking programs (smoke-free hospitals, helping healthcare workers quit, and counselling smoking patients).
- Smoking cessation should be promoted by strengthening the counselling network (overviewing and reforming the service, improving its marketing, and extending it to primary care physicians and dentists).

Since 2005, the National Health Insurance Fund has financed smoking cessation support in OPCs, although at a low value^35^. Varenicline has been available since 2007, although without reimbursement, similar to other drugs used to treat withdrawal symptoms^36^. In line with the WHO FCTC, experts have suggested that a public health product tax be imposed on tobacco products to finance the cost of cessation support; however, this initiative has not gained ground^37^.

In 2009, the Center for a Healthy Hungary established a quitline service based on a grant from a pharmaceutical company and its foundation. It was also tasked with training experts in cessation counselling^38^.

### The founding of the National Methodology Center for Smoking Cessation Support

In 2012, the National Methodology Center for Smoking Cessation Support (Center) was established at the NKIP owing to a European Union project grant (Social Renewal Operative Program - TÁMOP 6.1.2-11/4-2012-0001; active period: 2012–2014; maintenance period: 2015–2018) and was trusted with the following tasks to oversee and coordinate smoking cessation activities in Hungary:

- Education of healthcare workers in brief intervention and behavioral counselling;
- Operation of the national telephone counselling service;
- Formulation and regular updates of smoking cessation guidelines;
- Coordination of cessation support programs and cooperation in tobacco control activities;
- Integration of the course of cessation support in the continuing education of primary care physicians; and
- Continuously review and develop center operations.

The Center’s staff comprises physicians, psychologists, health promotion, and communication specialists. Since 2014, the Center’s operation has been financed annually by the state budget.

*Education and specialist training*

During the active period of the TÁMOP project (2012–2014), 240 healthcare professionals, mainly pulmonologists, OPC nurses, and psychologists, were trained in cognitive behavioral therapy counselling, and 437 were educated in brief intervention.

From 2014 to 2019, the Center participated in the Eastern Europe Nurses’ Center of Excellence for Tobacco Control (EE-COE) project coordinated by the Czech organization Society for Treatment of Tobacco-Dependence (STTD) in collaboration with the International Society of Nurses in Cancer Care (ISNCC), with expert support from the Schools of Nursing, University of California, Los Angeles, and University of California, San Francisco^39^. The project aimed to expand efforts to build nursing capacity to address tobacco dependence in Central and Eastern Europe. In Hungary, the project significantly improved the education of nurses and other healthcare professionals, such as health visitors and health promotion specialists, in the practice of brief intervention and advocacy in tobacco control.

Apart from the EE-COE project, the decision by the Secretary of Healthcare in 2020 that every health promotion office (HPO, a network of institutions supporting the development of health awareness) must employ a trained cessation support counsellor, reinforced the training of professionals. As a result, in 2014–2023, 436 physicians, nurses, health visitors, and health promotion specialists received training in cognitive behavioral therapy counselling and 1404 healthcare workers in brief intervention. Trained counsellors are offered regular supervision sessions to discuss recruitment and counselling challenges, share ideas, and define areas for training improvement.

Altogether, since the establishment of the Center, 676 healthcare professionals have received training in cognitive behavioral therapy counselling and 1841 in brief intervention.

In 2020–2023, the lecturers of the public health departments of the four Hungarian medical universities completed the Center’s cognitive behavioral therapy training. They included the practice of cessation support in the curricula of medical students and those participating in health science courses. Several nursing schools have also begun to include cessation support in their curriculum.

*Different forms of cessation support*

The TÁMOP project expected group counselling from the involved OPCs, which, being a firmly structured process, requires a high level of motivation to quit smoking and a lot of effort and organization from the provider; thus, it overburdened the OPCs. During the active period of the project, 5138 smokers were recruited in group counselling in 78 OPCs.

Since the end of the active period of the TÁMOP project, the number of smokers treated at OPCs (currently, there are 147 OPCs functioning) has remained relatively steady (on average, 5200 smokers per year) until 2020, when, most likely due to the COVID-19 pandemic, it decreased (to an average of 1500 smokers per year). However, the extent of treatment is unknown because there are insufficient data on whether smokers received only brief interventions or participated in behavioral counselling^40^.

Since 2020, cessation support counselling has gradually shifted from OPCs to HPOs. The population has broad access to cessation support services, as counselling is available in 92% of the 116 functioning HPOs^41^. However, a long-term funding system for cessation programs, including nicotine withdrawal treatment and communication, is inevitable and is yet to be established. Additionally, according to the guidelines on smoking cessation support, tobacco use, brief intervention, and behavioral counselling data should be interconnected between healthcare providers and cessation support services to monitor counselling activity and adjust programs to the population’s needs^42^. Currently, no registry records brief intervention practice, referral to behavioral counselling, or the practice and outcome of cessation support activities.

In addition to the mandatory health warnings in pictures and text that must be incorporated into tobacco product packaging according to the EU Tobacco Products Directive, in line with the Hungarian legislation, tobacco packaging and no-smoking signs must also contain information on assistance in quitting, such as a phone number – one of the possible endpoints being the national telephone counselling service – and a website^43^. Interest in the telephone counselling service is steady (on average, 1000 incoming calls per year), except for campaign periods when the number of smokers seeking help rises. Approximately 40% of callers agree to participate in the program, which could be increased with targeted public awareness campaigns and more frequent referrals of motivated individuals by healthcare professionals^44^. Although telephone counselling is a well-developed behavior change program that can help motivated individuals quit, it is insufficient in cases of high nicotine dependence. Consequently, the Center aims to promote telephone counselling services among the population and healthcare professionals with whom the Center can cooperate in building motivation for smokers and aiding the process of quitting smoking.

In 2019, the Center developed the ‘Facing a problem? Don’t reach for the stick!’ mobile phone application, designed to help smokers maintain their motivation by providing an overview of how their health improves with their efforts to stop smoking and showing how much money they have saved since their last cigarette. In 2022, the app was upgraded with the ‘21-day quit challenge’ program, offering daily guidance and tasks to smokers motivated to quit but would instead try to quit on their own rather than with the help of a counsellor^45^. The first evaluations of the program showed that even though participants indicated a strong motivation to quit at the start of the program, it was frequently interrupted, and the challenge was not completed in most cases. This may be explained by the fact that the users of the application were moderately nicotine-dependent and had a significant smoking history, thus requiring more support. As smoking cessation guidelines indicate, mobile phone and internet-based interventions are recommended to be used in combination with other counseling-based intervention strategies^46^. Although it is not known whether app users took a drug to treat withdrawal symptoms, it is important to pay more attention to pharmacotherapy as well during all quit attempts.

### Prospects for smoking cessation support services

In 2018–2019, the Center organized a 17-month cessation support counselling program in OPCs to determine the conditions to increase the effectiveness and efficiency of cessation support work, the direction of the development of this activity, and the training of cessation support counsellors^47^. The results of the program support the following conclusions:

1. Quitting smoking is more successful with a shorter smoking history^4,47^. Therefore, the critical task of health education is to reach smokers as early as possible and encourage them to quit.
2. It is essential that those smokers begin the behavioral counselling program who have already decided to quit firmly intend to participate and are therefore more committed to changing their lifestyle^4,47^.
3. It is essential to maintain motivation because, despite a firm intention to quit, most smokers cannot assess the challenges associated with quitting in advance^4^. The initial firm intention to quit does not guarantee full participation in counselling sessions, so the difficulties associated with quitting can lead to giving up^47^. It is recommended that the intention and motivation to quit smoking be strengthened at each consultation during routine medical care^4^.
4. More attention must be paid to the appropriate use of drugs to treat withdrawal symptoms during counsellor training and cessation support counselling. Pharmacotherapy helps during the initial period of quitting, but in the long-term, emphasis should be placed on mental and emotional factors and behavioral changes^47^.
5. Long-term support, including cognitive behavioral therapy and pharmacotherapy, led and supervised by a specialist in cessation counselling, contributes to more effective long-term abstinence^4,46,47^.
6. Providing counselling in the offices of medical providers during routine work hours (such as in OPCs) requires adequate funding, a disproportionate amount of organization, and flexibility of the provider and the smoker, which may reduce the willingness to join a counselling program^47^. Cessation support counselling services should operate as an independent specialist care. They must function in connection with frequently visited outpatient and inpatient healthcare services with adequate funding and trained professionals dedicated to cessation support^46,47^.

## CONCLUSION

The present narrative review brings forward a historical depiction of smoking cessation support in Hungary. The scientific community’s understanding of the problem of tobacco use has evolved dramatically over the past century, and best practice modalities of cessation support counselling have been available since the 1960s. However, the lack of adequate funding, as is the case in Hungary, for cessation programs that finance counselling services and treatment for nicotine withdrawal stops the progression of tobacco control. Long-term, adequate, and sustained financing should be available for tobacco control programs, such as cessation support counselling services and organizations responsible for tobacco control. Furthermore, the role of healthcare professionals in reducing the burden of the tobacco epidemic is outstanding, therefore, their individual responsibilities must be recognized. Despite the many healthcare professionals trained in brief intervention and cognitive behavioral therapy in Hungary, patient motivation and referral to intensive counselling are insufficient and must be embedded in daily routine work. This requires prioritizing health awareness among the population and healthcare professionals through professional communication and campaigns.

## Data Availability

Data sharing is not applicable to this article as no new data was created.
